# Combination of plasma amyloid beta_(1-42/1-40)_ and glial fibrillary acidic protein strongly associates with cerebral amyloid pathology

**DOI:** 10.1186/s13195-020-00682-7

**Published:** 2020-09-28

**Authors:** Inge M. W. Verberk, Elisabeth Thijssen, Jannet Koelewijn, Kimberley Mauroo, Jeroen Vanbrabant, Arno de Wilde, Marissa D. Zwan, Sander C. J. Verfaillie, Rik Ossenkoppele, Frederik Barkhof, Bart N. M. van Berckel, Philip Scheltens, Wiesje M. van der Flier, Erik Stoops, Hugo M. Vanderstichele, Charlotte E. Teunissen

**Affiliations:** 1 Neurochemistry Laboratory, Department of Clinical Chemistry, Amsterdam Neuroscience, Vrije Universiteit Amsterdam, Amsterdam UMC, Boelelaan 1117, 1081 HV Amsterdam, The Netherlands; 2 Alzheimer Center Amsterdam, Department of Neurology, Amsterdam Neuroscience, Vrije Universiteit Amsterdam, Amsterdam UMC, Amsterdam, The Netherlands; 3 ADx NeuroSciences, Ghent, Belgium; 4 Department of Radiology and Nuclear Medicine, Amsterdam Neuroscience, Vrije Universiteit Amsterdam, Amsterdam UMC, Amsterdam, The Netherlands; 5 Clinical Memory Research Unit, Lund University, Lund, Sweden; 6 UCL Institutes of Neurology and Healthcare Engineering, London, UK; 7 Department of Epidemiology and Data Science, Vrije Universiteit Amsterdam, Amsterdam UMC, Amsterdam, The Netherlands; 8 Biomarkable, Ghent, Belgium

**Keywords:** Blood-based biomarkers, Plasma GFAP, Plasma amyloid beta, Amyloid pathology, Alzheimer’s continuum

## Abstract

**Background:**

Blood-based biomarkers for Alzheimer’s disease (AD) might facilitate identification of participants for clinical trials targeting amyloid beta (Abeta) accumulation, and aid in AD diagnostics. We examined the potential of plasma markers Abeta_(1-42/1-40)_, glial fibrillary acidic protein (GFAP) and neurofilament light (NfL) to identify cerebral amyloidosis and/or disease severity.

**Methods:**

We included individuals with a positive (*n* = 176: 63 ± 7 years, 87 (49%) females) or negative (*n* = 76: 61 ± 9 years, 27 (36%) females) amyloid PET status, with syndrome diagnosis subjective cognitive decline (18 PET+, 25 PET−), mild cognitive impairment (26 PET+, 24 PET−), or AD-dementia (132 PET+). Plasma Abeta_(1-42/1-40)_, GFAP, and NfL were measured by Simoa. We applied two-way ANOVA adjusted for age and sex to investigate the associations of the plasma markers with amyloid PET status and syndrome diagnosis; logistic regression analysis with Wald’s backward selection to identify an optimal panel that identifies amyloid PET positivity; age, sex, and education-adjusted linear regression analysis to investigate associations between the plasma markers and neuropsychological test performance; and Spearman’s correlation analysis to investigate associations between the plasma markers and medial temporal lobe atrophy (MTA).

**Results:**

Abeta_(1-42/1-40)_ and GFAP independently associated with amyloid PET status (*p* = 0.009 and *p* < 0.001 respectively), and GFAP and NfL independently associated with syndrome diagnosis (*p* = 0.001 and *p* = 0.048 respectively). The optimal panel identifying a positive amyloid status included Abeta_(1-42/1-40)_ and GFAP, alongside age and APOE (AUC = 88% (95% CI 83–93%), 82% sensitivity, 86% specificity), while excluding NfL and sex. GFAP and NfL robustly associated with cognitive performance on global cognition and all major cognitive domains (GFAP: range standardized β (sβ) = − 0.40 to − 0.26; NfL: range sβ = − 0.35 to − 0.18; all: *p* < 0.002), whereas Abeta_(1-42/1-40)_ associated with global cognition, memory, attention, and executive functioning (range sβ = 0.22 – 0.11; all: *p* < 0.05) but not language. GFAP and NfL showed moderate positive correlations with MTA (both: Spearman’s rho> 0.33, *p* < 0.001). Abeta_(1-42/1-40)_ showed a moderate negative correlation with MTA (Spearman’s rho = − 0.24, *p* = 0.001).

**Discussion and conclusions:**

Combination of plasma Abeta_(1-42/1-40)_ and GFAP provides a valuable tool for the identification of amyloid PET status. Furthermore, plasma GFAP and NfL associate with various disease severity measures suggesting potential for disease monitoring.

## Introduction

Alzheimer’s disease (AD) is a multifactorial disease, with amyloid beta (Abeta) accumulation in the brain as one of the first detectable pathological hallmarks [1–3] in concert with accumulating tau pathology, neuronal damage, synapse loss, and inflammation [1]. Amyloid pathology can be identified in vivo with positron emission tomography (PET) scans or through altered biomarker levels in the cerebrospinal fluid (CSF) [3–5]. Given the costs of PET and the invasiveness of CSF analysis, blood-based biomarkers accurately reflecting AD pathological processes are urgently needed. Such biomarkers will facilitate the identification and selection of participants for disease modifying clinical trials (e.g., targeting Abeta accumulation) and could help in monitoring of disease progression or therapeutic effectiveness.

With the establishment of new, sensitive analytical techniques, recent studies showed promising results for plasma Abeta as biomarker of ongoing amyloid pathology [6–17]. Particularly, results obtained with (semi-)automated platforms with high-throughput such as the Single Molecule Array (Simoa) technology [18] are promising, since implementation of these platforms into clinical practice is fairly straightforward. Using Simoa, individuals with evidence of ongoing amyloid pathology can be discriminated from those without with reasonable diagnostic accuracy (ranging between 68 and 79%) [9–11, 14–16], even in the pre-symptomatic phase [10, 11, 16]. In view of the multiple aspects of AD pathology, establishing a blood-based biomarker panel combining several markers is likely needed to further increase the diagnostic accuracy by measuring the complexity of AD pathology comprehensively. Moreover, having a combination of markers that not only reflects amyloid accumulation but also reflects the extent of neurodegeneration might allow for use as disease severity and therapeutic effectiveness monitoring tool. In this respect, neurofilament light (NfL) and glial fibrillary acidic protein (GFAP) are promising blood-based biomarkers. NfL reflects axonal damage [19, 20] and studies convincingly show that across the AD continuum NfL levels increase in serum and plasma [20–23]. Moreover, NfL levels were higher in non-demented individuals with evidence of amyloid pathology compared to those without [21, 24], and NfL levels increase already in the pre-symptomatic Alzheimer’s disease stages [25], with an accelerated increase at time of symptom onset [26]. GFAP reflects reactive astrocytosis and a recent study showed that serum GFAP levels were higher in AD patients compared to controls [27], in line with findings in the CSF [27–31]. Moreover, pathology studies showed that GFAP expression was higher in areas surrounding Abeta plaques, even in the earliest stages, [32–34] and expression levels increased along with the progression of tau pathology [33, 35].

In the current study, we aimed to investigate the utility of the combination of plasma biomarkers Abeta_(1-42/1-40)_, GFAP, and NfL, all measured on a single platform (Simoa), to identify AD pathology as determined with amyloid PET in a total of 252 individuals across the clinical AD spectrum, i.e., subjective cognitive decline (SCD), mild cognitive impairment (MCI), or AD-dementia. In addition, we investigated their potential to measure disease severity by evaluating associations with syndrome diagnosis and neuropsychological and imaging measures.

## Methods

### Study population

This study included 252 subjects from the Amsterdam Dementia Cohort [36, 37] with a baseline syndrome diagnosis of SCD (*n* = 70), MCI (*n* = 50), or AD-dementia (*n* = 132) with an amyloid PET scan available within 1 year from baseline diagnosis. Additionally, a plasma EDTA sample had to be available in the Amsterdam dementia biobank, which was collected within one year from both the clinical diagnosis and the amyloid PET scan. Research was approved by the medical ethical committee of the VU University medical center and was in accordance with the Helsinki Declaration of 1975. All subjects provided written informed consent to use medical data and biomaterials for scientific research.

Subjects visited the Alzheimer center Amsterdam between November 2008 and October 2018 for standardized dementia screening consisting of neurological, physical, and neuropsychological evaluation and brain magnetic resonance imaging (MRI) [36, 37]. Diagnoses were made in a multidisciplinary consensus meeting according to the then applicable guidelines [38–42]. The label of SCD was assigned when no abnormalities were observed on clinical or cognitive tests and when the criteria for MCI, dementia, or other medical conditions and psychiatric disorders that could potentially cause cognitive deficits were not met [38]. All AD dementia patients were required to have a positive amyloid PET scan [3].

### Cognitive assessment

Cognitive performance was assessed at dementia screening using a standardized neuropsychological test battery [37] covering global cognition and the four major cognitive domains memory, language, attention, and executive functioning. Global cognition was assessed by the Mini-Mental State Examination (MMSE). Memory was assessed by the Dutch version of the Rey Auditory Verbal Learning Test (RAVLT) with immediate recall, delayed recall, and recognition, and the Visual Association Test version A (VAT A; sum of trials 1 and 2). Language was assessed by the VAT-A naming and category fluency (animals). Attention was assessed by the Digit Span Forward, Trail Making Test (TMT) A, Stroop word naming, and Stroop color naming. Executive functioning was assessed by the Digit Span Backwards, TMT B, Stroop color word naming, and letter fluency (D-A-T). We applied a multiple imputation approach by creating fifteen imputed datasets to fill individual missing neuropsychological test scores (data availability ranged between 73 and 98%). Subsequently, TMT A, TMT B, and Stroop scores were inverted so that a lower score implicated worse performance for all administered tests. Next, all test scores were transformed into *Z* scores and domain scores were calculated by averaging the *Z*-transformed individual neuropsychological test scores. Analyses were performed on the pooled datasets.

### MRI

For *n* = 182 (72%), a visual rating of medial temporal lobe atrophy (MTA) according to the MTA-scale (range 0–4) [43] was performed. Right and left hemispheres were rated and subsequently averaged into one combined MTA score. MRI scans were acquired on 1.5 T MRI scanners (Sonata and Impact, Siemens, Germany; Signa HDXT, GE Healthcare, USA) or 3T MRI scanners (Discovery MR750 and Signa, GE Medical Systems, USA; Ingenuity TF PET/MR, Philips Medical Systems, the Netherlands; Titan, Toshiba Medical Systems, Japan).

### Amyloid PET

All subjects underwent an amyloid PET scan with [^18^F]florbetaben (*n* = 133), [^18^F]flutemetamol (*n* = 37), [^18^F]florbetapir (*n* = 33), or [^11^C]Pittsburgh compound-B (PiB; *n* = 49) tracers as part of research, on the PET/MR and Gemini TF-64 PET/CT scanner (Philips Medical Systems, The Netherlands) or on ECAT EXACT HR+ scanner (Siemens/CTI, Tennessee, USA). Procedures have been described in more detail elsewhere [44–48]. Scans were rated by an experienced nuclear medicine physician (BvB) as positive or negative for the presence of fibrillar amyloid pathology in the neocortex according to the guidelines of the tracer manufacturers. Briefly, interpretation of the images is performed visually. To do this, the activity in cortical grey matter is compared with activity in adjacent white matter. The negative scan has a typical white matter pattern. In the positive scan, the tracer signal in cortical regions is approximately similar to or higher than the signal in the adjacent white matter. In addition, in a positive scan there is a sharp contrast between the cortex and the skull, while in a negative scan this difference is more gradual. Five regions are rated on each scan, and if any of these five regions was clearly positive, then the image was classified as positive. A negative scan indicates no or sparse density of Abeta neuritic plaques in the brain, and a positive scan indicates a moderate to frequent amyloid plaque density. Our nuclear medicine physician (BvB) has a 100% intra-rater reliability between tracers within one subject.

### APOE genotyping

For *n* = 244 (97%), apolipoprotein E (APOE) genotyping was available. Sequencing was performed in EDTA plasma using Sanger sequencing on ABI130XL, after DNA amplification by PCR technique and analysis for size and quantity by QIAxcel DNA Fast Analysis kit. APOE ε4 carriers had one or two APOE ε4 copies, whereas non-carriers only carried APOE ε2 or APOE ε3 alleles.

### Simoa plasma analyses

EDTA plasma was collected through venipuncture under non-fasting conditions. Within 2 h of collection, plasma was centrifuged for 10 min at 1800×*g* at room temperature and stored at -80 °C in the Amsterdam dementia biobank in aliquots of 500 μL in 1.5/2-mL polypropylene tubes (Sarstedt, Germany). Prior to analysis, samples were thawed at room temperature using a cold-air fan, centrifuged at 10.000×*g* for 10 min and subsequently kept on ice until analysis. All samples were measured in duplicates onboard of the automated Simoa HD-1 analyzer by trained personnel.

Prototype assays were developed in-house that specifically detect Abeta _(1-42)_ and Abeta _(1-40)_, followed by transfer to ADx NeuroSciences (Ghent, Belgium) for further fine-tuning, upscaling, and adaptation of the assays to manufacturing conditions (named Amyblood). In short, the automated two-step analytical Simoa procedure of the Amyblood singleplex assays is as follows: in step one, for 120 min, 25 μL of 250 K helper beads (Quanterix, USA) and 250 K paramagnetic carboxylated beads that were activated with 0.1 mg/mL EDC (Thermo Scientific, USA) and coated with 0.2 mg/mL of either monoclonal antibody ADx102 (21F12) (for Abeta_(x-42)_ capture; ADx NeuroSciences) [49] or ADx103 (2G3) (for Abeta_(x-40)_ capture; ADx NeuroSciences) [49] are incubated with 100 μL of 4-fold (for Abeta_(1-42)_) or 10-fold (for Abeta_(1-40)_) pre-diluted plasma EDTA in PBS-based buffer with stabilizing protein, 200 μg/mL HBR-1 (Scantibodies Laboratory Inc., USA) and Tween 20 detergent, and with 20 μL of 0.1 μg/mL of biotinylated monoclonal antibody ADx101 (3D6) (for Abeta_(1-x)_ detection; ADx NeuroSciences) [49]. After a wash cycle, a 5-min and 15-s incubation step followed with 50 pM streptavidin-conjugated β-galactosidase (Quanterix). After the next wash, 25 μL Resorufin β-D-galactopyranoside (Quanterix) was added and beads are pulled onto the imaging disc, followed by time-lapsed fluorescent imaging. The standard curves were constructed in the range 0 to 64 pg/mL using Abeta_(1-40)_ and Abeta_(1-42)_ recombinant peptides (ADx NeuroSciences). A none-weighted, 4PL-fit algorithm was used to calculate sample concentrations in pg/mL.

For Amyblood analyses, Abeta_(1-42)_ and Abeta_(1-40)_ measurement occurred in sequential order within the same run. Abeta _(1-42)_ concentrations are expressed as ratio to Abeta _(1-40)_ (further referred to as: Abeta_(1-42/1-40)_). Good average intra-assay coefficients of variation (CV) of duplicate concentrations were obtained: 3% for Abeta _(1-42)_ and 2% for Abeta _(1-40)_. Average inter-assay %CV of the concentrations of three independent EDTA plasma pool quality controls measured over the runs was 14% for Abeta_(1-42)_ and 13% for Abeta_(1-40)_.

Plasma NfL and plasma GFAP were measured next in the same aliquot (introducing an additional freeze-thaw cycle). The commercially available Simoa™ NF-Light Advantage Kit (Quanterix) and Simoa™ GFAP Discovery Kit (Quanterix) were used according to manufacturer’s instructions and with on-board automated sample dilution. Good average intra-assay %CV of 5% for plasma NfL and 4% for GFAP were obtained. Average inter-assay %CV of the concentrations of three independent EDTA plasma pool quality controls measured over the runs was 2% for NfL and 8% for GFAP.

### Statistical analysis

Data were analyzed using SPSS for Windows version 22 (IBM) and graphs were constructed using R version 3.4.2. *P* < 0.05 was considered significant. Plasma biomarkers Abeta _(1-40)_, NfL and GFAP were right-skewed; thus, natural log (Ln) transformation was performed prior to statistical analyses. *Z*-transformation was performed on inverted Abeta_(1-42/1-40)_ and on Ln(GFAP) and Ln(NfL) when comparability of effect sizes was required.

We compared baseline characteristics between amyloid PET-positive and amyloid PET-negative individuals using chi-squared tests, *T* tests, or non-parametric equivalents. We used age- and sex-adjusted two-way ANOVA to assess the effects of amyloid PET status and syndrome diagnosis on plasma biomarker levels. Receiver operating characteristic (ROC) curves identifying positive amyloid PET scans were constructed and Youden’s cutoffs were calculated as the maximum sum of sensitivity and specificity. Using a backward elimination logistic regression procedure among all plasma markers, age, sex, and APOE ε4 carriership based on Wald’s *p* statistics, a panel that optimally identifies a positive amyloid PET status was generated. The logistic regression formula of the optimal panel with age entered as dichotomous variable (split on cohort’s average age) was calculated, to construct heat plots visualizing amyloid positivity screening capacity. Since AD disease-modifying clinical trials target to include the earliest disease stages, we additionally repeated the ROC analyses and logistic regression analysis restricted to the persons without dementia (SCD and MCI). As a sensitivity analysis to verify the selected panel, we applied Least absolute Shrinkage and Selection Operator (LASSO) regression both on the total study cohort and the non-demented subset (R package glmnet). We used a maximum of iteration of 1000 and selected a robust lambda (i.e., largest value of the lambda is within one standard error of the minimum). Relationships between plasma biomarkers and cognitive performance (as surrogate measure of disease severity) were assessed using linear regression analysis, adjusted for age, sex, and education (according to Verhage system [50]). Relationships between plasma biomarkers and the MTA score (as surrogate measure of disease severity) were assessed using Spearman’s rank correlation analysis.

## Results

### Cohort characteristics

Demographics and clinical characteristics are listed in Table 1 and supplementary Table 1. The study cohort comprised 176 (70%) amyloid PET positive and 76 (30%) amyloid PET negative individuals. The amyloid PET positive group comprised 18 individuals with syndrome diagnosis SCD, 26 with MCI, and 132 with AD dementia. The amyloid PET negative group comprised 52 individuals with SCD and 24 with MCI. There were less males in the amyloid PET positive group compared to the amyloid PET negative group (*p* = 0.042). Following expectations, MMSE and APOE ε4 carriership was distributed differently between the PET groups (both *p* < 0.001).

**Table 1:** Demographics, clinical characteristics, and plasma marker concentrations of the total study population and stratified for amyloid PET status

		Stratified for amyloid PET status	
	Total	Amyloid negative	Amyloid positive
	***n = 252***	***76 (30%)***	***176 (70%)***
Age	63 ± 8	61 ± 9	63 ± 7
Female sex	114 (45%)	27 (36%)	87 (49%) *
Education	5.3 ± 1.2	5.5 ± 1.3	5.2 ± 1.2
Syndrome diagnosis (SCD/MCI/AD-dementia)	70/50/132	52/24/0	18/26/132 **
APOE ε4 carriership	134 (53%)	18 (24%)	116 (66%) **
MMSE	24 ± 4	27 ± 2	23 ± 4 **
MTA score	1 (0–1.5)	0.5 (0–1)	1 (0.5–1.5) **
Plasma Abeta_(1-42/1-40)_	0.15 ± 0.03	0.17 ± 0.03	0.14 ± 0.03 *
Plasma Abeta_(1-42)_, pg/mL	24 ± 6	27 ± 6	23 ± 6 *
Plasma Abeta_(1-40)_, pg/mL	160 ± 29	165 ± 30	157 ± 28 *
Plasma GFAP, pg/mL	146 ± 78	96 ± 53	168 ± 77 **
Plasma NfL, pg/mL	14 ± 9	11 ± 6	15 ± 10

Baseline features of the total study population and stratified for visually read amyloid PET status is presented as mean ± SD, median (25th–75th percentile) or *n* (%). Education scoring is according to the Verhage (1965) system with a scale ranging from 1 to 7. Demographic and clinical differences between the two groups were calculated using independent *t* tests, chi-square tests, or Mann-Whitney *U* test as appropriate. Differences between plasma biomarker levels were calculated using two-way ANOVA for PET status and syndrome diagnosis adjusted for age and sex, of which the *p* value of the independent effect of PET status is presented here. Raw plasma biomarker values are presented in the table, but prior to statistical analysis Abeta_(1-40)_, NfL and GFAP were natural log-transformed for normality of the data. APOE status was available for *n* = 244, MTA score (average of right and left) was available for *n* = 182, plasma Abeta_(1-42/1-40)_ and Abeta_(1-42)_ for *n* = 238, plasma Abeta_(1-40)_ for *n* = 240, plasma GFAP for *n* = 247, and plasma NfL for *n* = 251

*PET* positron emission tomography, *SCD* subjective cognitive decline, *MCI* mild cognitive impairment, *AD* Alzheimer’s disease, *APOE* apolipoprotein E, *MMSE* mini mental state examination, *MTA* medial temporal lobe atrophy, *Abeta* amyloid beta, *GFAP* Glial fibrillary acidic protein, *NfL* Neurofilament light

*p < 0.05

**p < 0.001

### Plasma biomarkers in relation to amyloid PET status and syndrome diagnosis

**Fig. 1:**
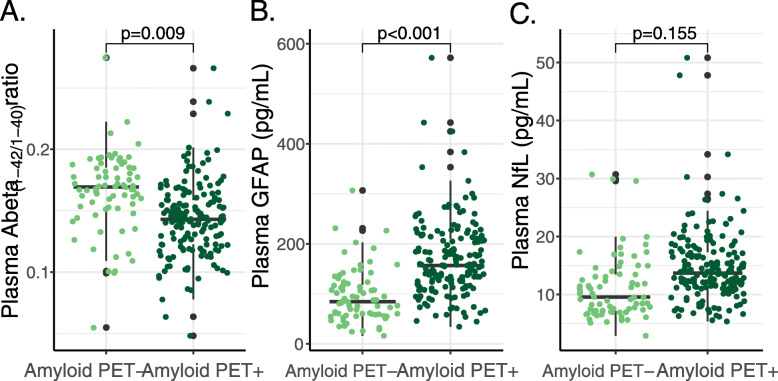
Boxplots of raw plasma biomarker levels for amyloid PET negative (−) and amyloid PET positive (+) individuals. Statistical analysis was conducted using age and sex adjusted two-way ANOVAs for amyloid PET status and syndrome diagnosis on the plasma biomarker levels, of which the *p* value of the independent effect of PET status is presented. Plasma GFAP and plasma NfL, levels were natural log transformed prior to statistical analysis. Abeta, amyloid beta; GFAP, glial fibrillary acidic protein; NfL, neurofilament light; PET, positron emission tomography

**Fig. 2:**
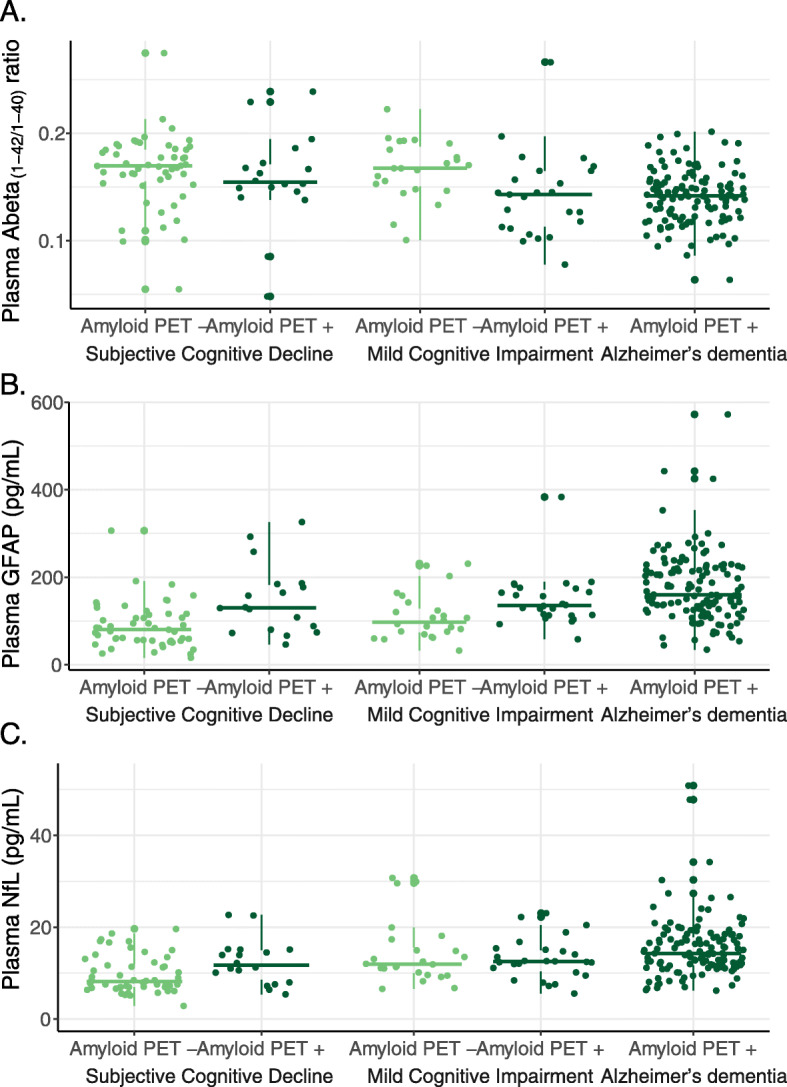
Boxplots of raw plasma biomarker levels for amyloid PET status (negative: -; positive: +) in function of the syndrome diagnostic groups. Statistical analysis was conducted using age and sex adjusted two-way ANOVAs evaluating the independent effects of amyloid PET status and syndrome diagnosis on the plasma biomarker levels. For plasma Abeta_(1-42/1-40)_, PET status had a main effect (*p* = 0009) but not syndrome diagnosis (*p* = 0.192). For GFAP, both PET (*p* < 0.001) and syndrome diagnosis (*p* = 0.048) had main effects. For NfL, syndrome diagnosis (*p* = 0.001) but not PET status (*p* = 0.155) had a main effect. Plasma GFAP and plasma NfL levels were natural log transformed prior to ANOVA. Abeta, amyloid beta; GFAP, glial fibrillary acidic protein; NfL, neurofilament light; PET, positron emission tomography

Age- and sex-adjusted two-way ANOVA showed a main effect of amyloid PET status (*F* = 6.996, *p* = 0.009) but not of syndrome diagnosis (*F* = 1.665, *p* = 0.192) for plasma Abeta_(1-42/1-40)_ level (Figs. 1a, 2a), indicating that brain amyloidosis leads to the decrease in plasma Abeta_(1-42/1-40)_ level. For plasma GFAP (Figs. 1b, 2b), there were main effects of both amyloid PET status (*F* = 21.307, *p* < 0.001) and syndrome diagnosis (*F* = 3.072, *p* = 0.048); thus, both brain amyloidosis and disease severity independently contribute to the increase in plasma GFAP level. For plasma NfL (Figs. 1c, 2c), there was a main effect of syndrome diagnosis (*F* = 6.823, *p* = 0.001) but not of amyloid PET status (*F* = 2.033, *p* = 0.155); thus, mainly disease severity leads to the increase in plasma NfL level. There were no interactions between amyloid PET status and syndrome diagnosis for any of the plasma markers.

### Diagnostic accuracy of plasma biomarkers in identifying a positive amyloid PET status

**Fig. 3:**
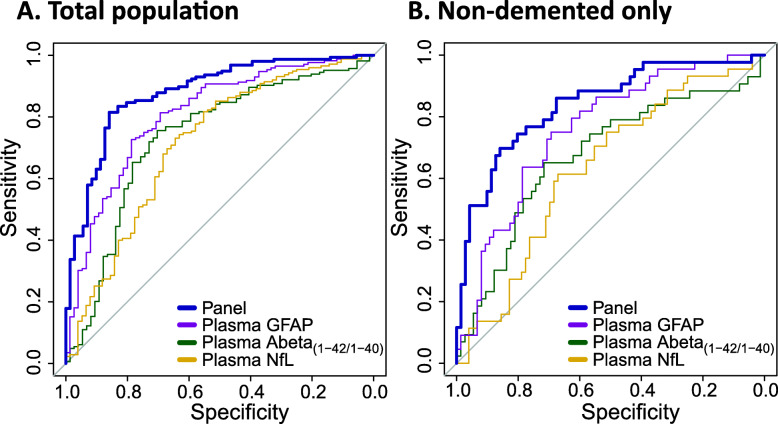
ROCs for amyloid PET positivity in the total study population (**a**) and non-demented subset (**b**). Individual plasma biomarkers GFAP, Abeta_(1-42/1-40)_, and NfL are plotted as well as the combined panel best predicting amyloid PET positivity. Panel in the total population (**a**) are the predicted values of the combined plasma Abeta_(1-42/1-40)_, plasma GFAP, age, and APOE ε4 carriership panel (AUC = 0.88 (95% CI 0.83–0.93)). Panel in the non-demented population (SCD + MCI) (**b**) are the predicted values of the combined plasma Abeta_(1-42/1-40)_, plasma GFAP, and APOE ε4 carriership panel (AUC = 0.84 (95% CI 0.76–0.92)). GFAP, glial fibrillary acidic protein; Abeta, amyloid beta; NfL, neurofilament light

ROC analysis for evaluating the correspondence of the plasma biomarkers to a positive amyloid PET status showed that all plasma markers individually were associated with amyloid PET positivity with AUC > 71% (Table 2, Fig. 3a). At the cutoff optimized for balanced sensitivity and specificity (Youden’s Index), the sensitivity of all single markers was between 70 and 73%, whereas specificity was 76 and 79% for plasma Abeta_(1-42/1-40)_ and GFAP respectively, and only 64% for plasma NfL (Table 2). In comparison, APOE ε4 carriership was associated with amyloid PET positivity (AUC = 72% (95% CI 66–79%); sensitivity 68%, specificity 76%)), while age and sex were not (age: AUC = 51% (95% CI 42–59%); sex: AUC = 57% (95% CI 49–65%)). To define the panel that optimally identifies a positive amyloid PET scan, we used Wald’s backward elimination logistic regression analysis among all plasma markers and APOE, age, and sex. The optimal panel included the variables plasma Abeta_(1-42/1-40)_ and plasma GFAP, alongside age and APOE, and reached an AUC of 88% (95% CI 83–93%; Table 2; Fig. 3a). The positive predictive value (PPV) of this panel was 93% and the sensitivity was 82% (negative predictive value (NPV): 68%, specificity: 86%).

**Table 2:** AUC and sensitivity and specificity at Youden’s cutoff to identify an abnormal amyloid PET scan in the total study population and in the non-demented subset

	AUC (95% CI)	Youden’s cut-point	Sensitivity (%)	Specificity (%)
**Total population**				
Plasma Abeta_(1-42/1-40)_	73% (66–81%)	0.16	70	76
Plasma GFAP	81% (75–87%)	125 pg/mL	73	79
Plasma NfL	71% (64–79%)	11.5 pg/mL	73	64
Panel*	***88% (83–93%)***	***–***	***82***	***86***
**Non-demented subset (SCD + MCI)**				
Plasma Abeta_(1-42/1-40)_	67% (57–78%)	0.16	72	65
Plasma GFAP	76% (67–85%)	108 pg/mL	75	69
Plasma NfL	63% (53–73%)	11.9 pg/mL	61	67
***Panel***^***ǂ***^	***84% (76–92%)***	***–***	***70***	***86***

AUC with 95% confidence interval was calculated using receiver operator curve (ROC) analysis. Youden’s cut-point is at the coordinates of the ROC curve where a maximum sum of sensitivity and specificity is reached. For the single markers, this results in a useable cutoff thus presented here, whereas for the panels, this is a predicted value from the logistic regression model. The panels were established using an automated Wald’s backward selection procedure among plasma markers Abeta_(1-42/1-40)_, GFAP, NfL, age, sex, and APOE ε4 carriership. Predicted values of the logistic regression analysis are used for ROC analysis

*Abeta* amyloid beta, *GFAP* glial fibrillary acidic protein, *NfL* neurofilament light, *SCD* subjective cognitive decline, *MCI* mild cognitive impairment, *AUC* area under the curve, *95%CI* 95% confidence interval

*For the total population, the panel includes plasma Abeta_(1-42/1-40)_, plasma GFAP, APOE ε4 carriership, and age

^ǂ^For the non-demented subset (SCD and MCI), the panel includes Abeta_(1-42/1-40)_, plasma GFAP, and APOE ε4 carriership

We repeated the analysis restricted to the persons without dementia (SCD and MCI; Table 2). Comparable to the total population, in this non-demented subset we observed an association of APOE with amyloid PET positivity (AUC = 74% (95% CI 65–84%) while age and sex were not associated (age: AUC = 48% (95% CI 37–59%); sex: AUC = 55% (44–66%). AUCs of the single markers for amyloid PET positivity were slightly lower in the non-demented subset when compared to the total study population (largest difference in AUCs observed for plasma NfL, i.e., from 71% to 63%). Sensitivity of plasma Abeta_(1-42/1-40)_ and plasma GFAP in the non-demented subset were comparable to sensitivity observed in the total study population (Abeta_(1-42/1-40)_: 72%; GFAP: 75%), but sensitivity of NfL dropped to 61%. Also here, in Wald’s backward elimination logistic regression analysis among all plasma markers and APOE, age, and sex, the combination of plasma Abeta_(1-42/1-40)_, plasma GFAP and APOE was selected as the optimal panel, although age was not included. This panel resulted in an AUC of 84% (95% CI 76–92%; Fig. 3b), with a PPV of 75% and sensitivity of 70% (NPV: 82%, specificity 86%).

**Fig. 4:**
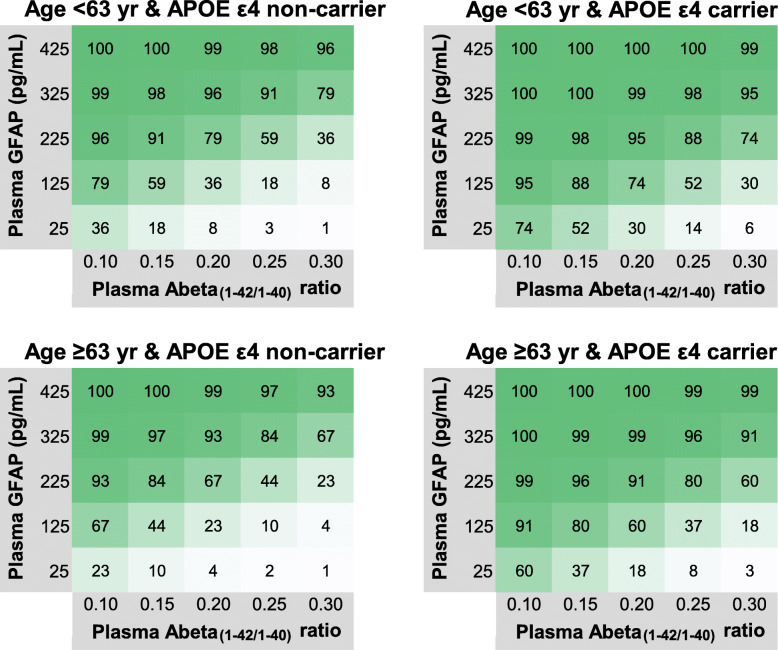
Heat plots with predicted probabilities for amyloid PET positivity in the total study population. Heat plots were constructed by filling out the logistic regression formula with constant = 0.839, and beta’s *B* = − 19.02 for Abeta_(1-42/1-40)_, *B* = 0.019 for GFAP, *B* = − 0.618 for age (dichotomous variable: younger (= 0) versus older (= 1) than cohort’s average age of 63 years) and *B* = 1.625 for APOE ε4 carriership (non-carrier = 0, carrier = 1). Abeta, amyloid beta; GFAP, glial fibrillary acidic protein; APOE, apolipoprotein E

To visualize how our panel could be operationalized in identifying a positive amyloid PET status, we constructed heat plots (Fig. 4) representing the percentage likelihood of having a positive amyloid PET status based on plasma Abeta_(1-42/1-40)_ and plasma GFAP levels, after stratifying for age (younger or older than total cohort’s average age of 63 years) and APOE ε4 carriership for the total study cohort (heat plots for the non-demented subset are presented in supplementary Figure 1). The plots illustrate that with decreasing Abeta_(1-42/1-40)_ in combination with increasing GFAP levels, the probability of being amyloid PET positive rises. The probabilities are in general higher in the APOE ε4 carriers when comparing to the non-carriers and are higher in the younger individuals when comparing to the older individuals. For example, an APOE ε4 carrier younger than 63 years old has a 88% risk of being amyloid positive when plasma GFAP is 125 pg/mL and plasma Abeta_(1-42/1-40)_ is 0.15, whereas this is 59% in the younger APOE ε4 non-carriers, 80% in the older (≥ 63 years) APOE ε4 carriers, and 44% in the older APOE ε4 non-carriers.

To verify the robustness of the selected panel, we performed LASSO regression analysis and observed that both in the total cohort and in the non-demented subset the variables Abeta_(1-42/1-40)_, GFAP and APOE ε4 carriership were selected. Contrary to the logistic regression analysis in the total study cohort, age did not contribute to the identification of a positive amyloid PET scan. In agreement with the logistic regression analyses, the LASSO regression confirmed that both Abeta_(1-42/1-40)_ and GFAP have independent predictive value for amyloid PET status, while NfL has not.

### Relationships between plasma biomarkers and disease severity measures

**Fig. 5:**
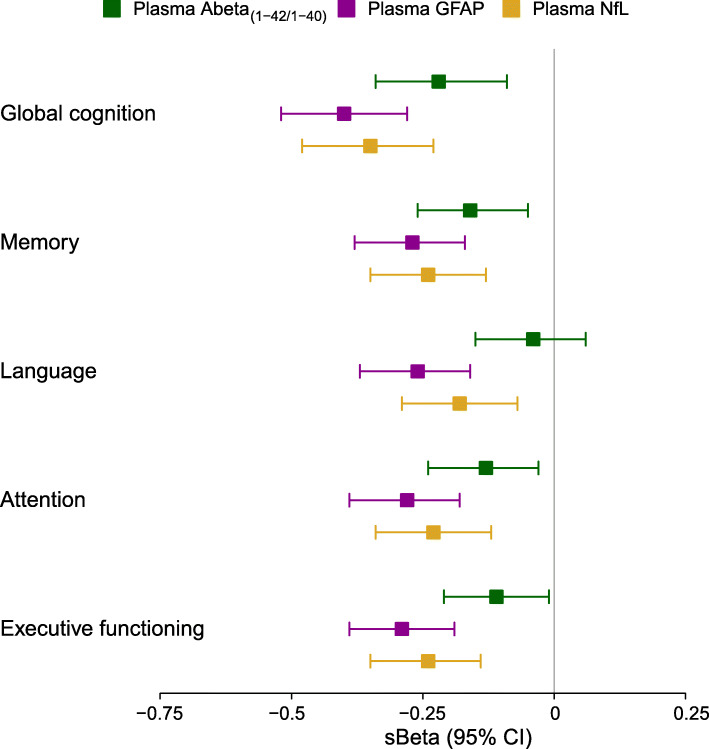
Associations of plasma biomarkers with cognitive performance across the total study cohort, presented as standardized effect sizes with 95% confidence intervals of age, sex, and education (according to Verhage (1965) system) adjusted linear regression analysis between plasma biomarker levels and cognitive domain scores. Plasma Abeta_(1-42/1-40)_ levels were inverted prior to analysis, so that the direction of effect sizes are comparable for all markers. Abeta, amyloid beta; GFAP, glial fibrillary acidic protein; NfL, neurofilament light

We explored the strength of relationships of our plasma biomarkers with disease severity by investigating their age, sex, and education-adjusted associations with cognitive performance (Fig. 5). Plasma GFAP and plasma NfL were robustly associated with global cognitive performance and cognitive performance in all major domains memory, language, attention, and executive functioning (GFAP: range standardized β (sβ) = − 0.40 to − 0.26; NfL: range sβ = − 0.35 to − 0.18; all: *p* < 0.05). Plasma Abeta_(1-42/1-40)_ was associated with global cognition, memory, attention, and executive functioning performance (range sβ = − 0.22 to − 0.11; all: *p* < 0.05), but not with language.

Using the MRI-based visual MTA scores as surrogate measure of disease severity, we observed a moderately strong positive correlation between plasma GFAP and MTA score (Spearman’s rho = 0.35, *p* < 0.001) and between plasma NfL and MTA score (Spearman’s rho = 0.33, *p* < 0.001). For plasma Abeta_(1-42/1-40)_, we observed a moderate negative correlation with MTA score (Spearman’s rho = − 0.24, *p* = 0.001).

## Discussion

In the present work, we showed that both plasma Abeta_(1-42/1-40)_ and plasma GFAP are independently associated with amyloid pathology as measured by PET in a cohort of individuals with SCD, MCI, and AD-dementia. Combining the plasma markers in a panel together with age and APOE yielded an accuracy for amyloid PET positivity of 88%, with a PPV of 93% and a sensitivity of 82%. The accuracy of this panel was comparable when the analysis was restricted to individuals without dementia (i.e., 84%), with PPV of 75% and sensitivity of 70%. The findings indicate that the combination of plasma Abeta_(1-42/1-40)_ and GFAP could be useful for the identification of clinical trial candidates that are progressing along the AD pathological continuum. Furthermore, plasma GFAP and NfL levels were quite robustly related to different disease severity measures (e.g., syndrome diagnosis, cognitive domain scores and MTA score), implying that plasma GFAP and NfL are promising putative biomarker as surrogate outcome measure in monitoring disease severity, and therapeutic effectiveness in clinical trials.

In line with expectations and consistent with multiple other recent reports [6–17], we found decreased plasma Abeta_(1-42/1-40)_ levels in amyloid PET positive individuals compared to amyloid negative individuals. Amyloid levels in blood are much lower when compared to amyloid levels in the CSF, and while amyloid levels in the CSF decrease by more than 50% upon brain amyloidosis, in the plasma this decrease is less than 20% [7]. Ultra-sensitive and robust blood amyloid measurement techniques are thus required to precisely detect the relatively small reduction in the already low amyloid blood levels between amyloid PET positive and negative individuals. In the last few years, new high-sensitive blood amyloid techniques have been developed, all having their own pros and cons in terms of technical capacities and clinical translatability. The Simoa technology has proven its sensitivity for the accurate detection of low-abundant analytes [18] and has the advantage that this method can be translated fairly straightforward into clinical practice due to its semi-automated procedure with relatively high throughput. In the current study, we applied Amyblood, our novel Simoa plasma amyloid assays that were developed using high-quality antibodies [49]. Our novel assays specifically measure the full-length Abeta isoforms 1-42 and 1-40, as compared to the current commercial Simoa assays that measures a mixture of full-length and n-truncated Abeta isoforms (i.e., n-42 and n-40). Measurement of the full-length Abeta isoforms only is preferred in, e.g., clinical trials, where it is fundamental to know what isoforms are detected in the blood in order to carefully map target engagement of therapeutics. We observed that the diagnostic accuracy of our Abeta_(1-42/1-40)_ measurement alone for amyloid PET positivity was reasonably good with an AUC of 73%. This accuracy is highly comparable to what was reported in another recent study that investigated Abeta_(1-42/1-40)_ in relation to brain amyloidosis, measured by another automated high through-put platform (i.e., AUC of 77%) [12].

Since the reduction in plasma amyloid levels upon brain amyloidosis is rather small, the simultaneous evaluation of other blood-based biomarkers might help in achieving a larger discriminative power between individuals with brain amyloidosis and those without. Additionally, a model containing a panel of markers probably better reflects the multifactorial nature of the Alzheimer’s disease pathophysiological changes therewith potentially increasing the sensitivity and specificity of an Alzheimer’s blood-based biomarker panel. Therefore, we measured GFAP and NfL levels, also using Simoa assays. Plasma GFAP proved particularly promising with a strong association with amyloid positivity (AUC = 81%), an independent contribution to a panel together with plasma Abeta_(1-42/1-40)_ and risk factors age and APOE (panel AUC = 88%), and the robust association with disease severity operationalized as cognitive performance in the major cognitive domains or MTA scores visually rated on MRI scans. Only one recent study described the potential of serum GFAP as biomarker for AD, by showing increased GFAP levels in CSF biomarker-confirmed AD-dementia cases compared to non-diseased controls [27], which was comparable to our findings and was in line with previous observations in the CSF of AD patients [27–31]. It is interesting to note that GFAP seemed to perform similarly well in identifying amyloid PET positivity as plasma Abeta_(1-42/1-40)_. GFAP is a marker of reactive astrocytes, the brain’s responders to various forms of injury including amyloid and tau aggregates [32–35]. It has additionally become apparent that reactive astrocytes might also be initiators, early modulators, or contributors to AD pathological progression, by acquiring neurotoxic functions upon morphological and functional changes that are driven by ongoing pathology [32, 34, 51]. Considering the diverse roles and effects of reactive astrocytes in AD, it is not that unexpected that we observed relatively strong relationships for plasma GFAP with both PET amyloid positivity as well as with clinical disease severity. Further validation of this blood-based biomarker is needed to further examine the utility of plasma GFAP for identifying a positive amyloid PET status, among others in a cohort in which non-AD dementia patients with amyloid co-pathology (e.g., Lewy body dementia) are included. The observed similar accuracy for plasma GFAP and plasma Abeta_(1-42/1-40)_ might also be analytically explained. GFAP might have more favorable characteristics for easier and reliable quantification as compared to Abeta_(1-42/1-40)_, since GFAP concentrations in blood are much higher. Furthermore, Abeta_(1-42/1-40)_ is a sticky and aggregation-prone protein of which the accurate and robust measurement both in plasma and CSF is influenced by pre-analytical sample handling factors [52–54]. Pre-analytical studies for all novel blood-based biomarkers will help further elucidating their diagnostic utility.

In addition to GFAP, we measured NfL as injury marker for AD and observed that NfL levels increase over the clinical spectrum from SCD to AD dementia. This finding is in agreement with various recent reports [20–23, 26, 55]. Adjusted for syndrome diagnosis, NfL did not relate to amyloid PET status and consequently did not have additional diagnostic value to Abeta_(1-42/1-40)_ and GFAP with age and APOE for the identification of amyloid PET positivity. Also, as surrogate outcome marker for disease severity, GFAP seemed to outperform NfL in our study. Since NfL is a product of brain injury (i.e., NfL disintegrates from the axon upon damage) and GFAP is a responder to both brain injury as well as amyloid and tau aggregates, we hypothesize that GFAP serves as a more sensitive marker for Alzheimer’s disease pathological processes.

When we studied the non-demented subset in more detail, we observed that the PPV and sensitivity of our panel were somewhat lower, although accuracy was comparable. Non-demented individuals with a positive PET scan are highly sought after for clinical AD prevention trials, and a good blood-based biomarker panel will facilitate their identification. Here, we measured multiple markers all assessed on a single technology. Further improvements for our panel could be inclusion of the emerging plasma pTau181 and pTau271 measurements [56, 57]. Another advantage of future addition of a pTau measure to our plasma panel would be that our panel then reflects the complete ATN (amyloid, tauopathy, neurodegeneration) classification [3], wherein “A” would be our Abeta_(1-42/1-40)_, “T” would be a pTau measure, and we would propose NfL or GFAP to measure “N.”

### Strengths

Among the strengths of our study is that we used a thoroughly characterized study population, including extensive cognitive performance data. Another strength is that our Amyblood assays that measure the full-length Abeta_(1-42)_ and Abeta_(1-40)_ isoforms using high-quality antibodies, as well as the other markers GFAP and NfL were all measured on the Simoa platform. This offers the opportunity to readily integrate all applied Simoa singleplex assays into one combined multiplex assay, which would increase the efficiency of measuring our proposed biomarker panel while simultaneously lowering the analyses costs and plasma volumes required. Follow-up of the study population is still ongoing, providing an opportunity to investigate longitudinal implications of our plasma markers in the future. Next studies that use longitudinal blood collection and longitudinal cognitive performance will help to fine-tune the monitoring potential of the plasma Abeta_(1-42/1-40)_ and GFAP panel.

### Limitations

Our study has some limitations as well. We chose to focus on the Alzheimer’s continuum, thereby excluding non-AD dementia patients. This resulted in a larger proportion of amyloid positive individuals as compared to what is seen in, e.g., clinical diagnostic practice, or in clinical trial settings where it is the goal to screen amyloid positivity preferably among non-demented individuals. The sample selection might have positively influenced the GFAP results, although when restricted to the non-demented subset, we maintained strong relationships of GFAP with amyloid PET status. It is to note that we did not include cognitively normal individuals without SCD in this study. Also, we only focused on a memory clinic-based cohort and did not include, e.g., a population-based cohort. These choices might have influenced our findings as well. As a next step, our findings should be validated in independent cohorts including cognitively normal individuals without SCD. Although, previous research showed that individuals with SCD that have a negative amyloid status are unlikely to show clinical progression to dementia [58], which provides confidence that our individuals with SCD are likely not in the prodromal stages of dementia and as such can be considered as normal controls. Another limitation is that our study cohort is relatively young which means they suffer less from comorbidities as compared to older populations. Results such as the presented heat plots may thus not be readily generalizable to other settings (e.g., to late-onset AD). Another limitation might be that PET scans were obtained with different tracers, although post-mortem studies have shown that different amyloid PET tracers have comparable sensitivity and specificity for amyloid pathology. Furthermore, all amyloid PET scans and MRI scans were only visually read. Even though quantitative approaches might generate somewhat different results, visual amyloid PET reads are the FDA-approved method of identifying amyloid positivity and as such is the method of reference. Moreover, all scans were read according to standardized procedures by one experienced nuclear medicine physician which increases the robustness of the visual readings. Due to the use of the different tracers, we did not investigate relationships between our plasma markers and amyloid load. Relationships between the plasma biomarkers and amyloid PET and MRI measures interpreted with quantitative approaches remains to be investigated in follow-up studies.

## Conclusions

To conclude, our results suggest that the combination of blood biomarkers Abeta_(1-42/1-40)_ and GFAP is useful to pre-screen for amyloid positivity. Additionally, GFAP and NfL are promising biomarker for monitoring of disease severity.

## Supplementary information

**Additional file 1: Supplementary Table 1.** Demographics, clinical characteristics, and plasma marker concentrations of the study population stratified by syndrome diagnosis. **Supplementary figure 1.** Heat plots with predicted probabilities for amyloid PET positivity in the non-demented subset.

## Data Availability

The datasets generated and/or analyzed during the current study are not publicly available due to local data storage but are available from the corresponding author upon request.

## Abbreviations

AD: Alzheimer’s disease
Abeta: Amyloid beta
PET: Positron emission tomography
CSF: Cerebrospinal fluid
NfL: Neurofilament light
GFAP: Glial fibrillary acidic protein
SCD: Subjective cognitive decline
MCI: Mild cognitive impairment
MRI: Magnetic resonance imaging
MMSE: Mini-Mental State Examination
RAVLT: Rey Auditory Verbal Learning Test
VAT: Visual Association Test
TMT: Trail Making Test
MTA: Medial temporal lobe atrophy
APOE: Apolipoprotein E

## References

[1] Bateman RJ, Xiong C, Benzinger TL, Fagan AM, Goate A, Fox NC (2012). Clinical and biomarker changes in dominantly inherited Alzheimer’s disease. N Engl J Med.

[2] Sperling RA, Aisen PS, Beckett LA, Bennett DA, Craft S, Fagan AM (2011). Toward defining the preclinical stages of Alzheimer’s disease: recommendations from the National Institute on Aging-Alzheimer’s Association workgroups on diagnostic guidelines for Alzheimer’s disease. Alzheimers Dement.

[3] Jack CR, Bennett DA, Blennow K, Carrillo MC, Dunn B, Haeberlein SB (2018). NIA-AA Research Framework: toward a biological definition of Alzheimer’s disease. Alzheimers Dement.

[4] Scheltens P, Blennow K, Breteler MM, de Strooper B, Frisoni GB, Salloway S (2016). Alzheimer’s disease. Lancet..

[5] Toledo JB, Zetterberg H, van Harten AC, Glodzik L, Martinez-Lage P, Bocchio-Chiavetto L (2015). Alzheimer’s disease cerebrospinal fluid biomarker in cognitively normal subjects. Brain..

[6] Nakamura A, Kaneko N, Villemagne VL, Kato T, Doecke J, Dore V (2018). High performance plasma amyloid-beta biomarkers for Alzheimer’s disease. Nature..

[7] Ovod V, Ramsey KN, Mawuenyega KG, Bollinger JG, Hicks T, Schneider T (2017). Amyloid beta concentrations and stable isotope labeling kinetics of human plasma specific to central nervous system amyloidosis. Alzheimers Dement.

[8] Nabers A, Perna L, Lange J, Mons U, Schartner J, Guldenhaupt J, et al. Amyloid blood biomarker detects Alzheimer’s disease. EMBO Mol Med. 2018;10(5):e8763. 10.15252/emmm.201708763 PMC5938617 29626112

[9] Janelidze S, Stomrud E, Palmqvist S, Zetterberg H, van Westen D, Jeromin A (2016). Plasma beta-amyloid in Alzheimer’s disease and vascular disease. Sci Rep.

[10] Verberk IMW, Slot RE, Verfaillie SCJ, Heijst H, Prins ND, van Berckel BNM (2018). Plasma amyloid as prescreener for the earliest Alzheimer pathological changes. Ann Neurol.

[11] Vergallo A, Megret L, Lista S, Cavedo E, Zetterberg H, Blennow K, et al. Plasma amyloid beta 40/42 ratio predicts cerebral amyloidosis in cognitively normal individuals at risk for Alzheimer’s disease. Alzheimers Dement. 2019;15(6):764–75. 10.1016/j.jalz.2019.03.009 31113759

[12] Palmqvist S, Janelidze S, Stomrud E, Zetterberg H, Karl J, Zink K, et al. Performance of fully automated plasma assays as screening tests for Alzheimer disease-related beta-amyloid status. JAMA Neurol. 2019;76(9):1060–9. 10.1001/jamaneurol.2019.1632 PMC6593637 31233127

[13] Shahpasand-Kroner H, Klafki HW, Bauer C, Schuchhardt J, Huttenrauch M, Stazi M (2018). A two-step immunoassay for the simultaneous assessment of Abeta38, Abeta40 and Abeta42 in human blood plasma supports the Abeta42/Abeta40 ratio as a promising biomarker candidate of Alzheimer’s disease. Alzheimers Res Ther.

[14] Shi Y, Lu X, Zhang L, Shu H, Gu L, Wang Z (2019). Potential value of plasma amyloid-beta, total tau, and neurofilament light for identification of early Alzheimer’s disease. ACS Chem Neurosci.

[15] Li WW, Shen YY, Tian DY, Bu XL, Zeng F, Liu YH (2019). Brain amyloid-beta deposition and blood biomarkers in patients with clinically diagnosed Alzheimer’s disease. J Alzheimers Dis.

[16] Chatterjee P, Elmi M, Goozee K, Shah T, Sohrabi HR, Dias CB (2019). Ultrasensitive detection of plasma amyloid-beta as a biomarker for cognitively normal elderly individuals at risk of Alzheimer’s disease. J Alzheimers Dis.

[17] Schindler SE, Bollinger JG, Ovod V, Mawuenyega KG, Li Y, Gordon BA, et al. High-precision plasma beta-amyloid 42/40 predicts current and future brain amyloidosis. Neurology. 2019;93(17):e1647–59. 10.1212/WNL.0000000000008081 PMC6946467 31371569

[18] Wilson DH, Rissin DM, Kan CW, Fournier DR, Piech T, Campbell TG (2016). The Simoa HD-1 analyzer: a novel fully automated digital immunoassay analyzer with single-molecule sensitivity and multiplexing. J Lab Autom.

[19] Khalil M, Teunissen CE, Otto M, Piehl F, Sormani MP, Gattringer T (2018). Neurofilaments as biomarkers in neurological disorders. Nat Rev Neurol.

[20] Bridel C, van Wieringen WN, Zetterberg H, Tijms BM, Teunissen CE, and the NFLG, et al. Diagnostic value of cerebrospinal fluid neurofilament light protein in neurology: a systematic review and meta-analysis. JAMA Neurol. 2019;76(9):1035–48. 10.1001/jamaneurol.2019.1534 PMC6580449 31206160

[21] Mattsson N, Andreasson U, Zetterberg H, Blennow K (2017). Alzheimer’s disease neuroimaging I. Association of plasma neurofilament light with neurodegeneration in patients with Alzheimer disease. JAMA Neurol.

[22] Lewczuk P, Ermann N, Andreasson U, Schultheis C, Podhorna J, Spitzer P (2018). Plasma neurofilament light as a potential biomarker of neurodegeneration in Alzheimer’s disease. Alzheimers Res Ther.

[23] Zhou W, Zhang J, Ye F, Xu G, Su H, Su Y (2017). Plasma neurofilament light chain levels in Alzheimer’s disease. Neurosci Lett.

[24] Hu H, Chen KL, Ou YN, Cao XP, Chen SD, Cui M (2019). Neurofilament light chain plasma concentration predicts neurodegeneration and clinical progression in nondemented elderly adults. Aging (Albany NY).

[25] Preische O, Schultz SA, Apel A, Kuhle J, Kaeser SA, Barro C (2019). Serum neurofilament dynamics predicts neurodegeneration and clinical progression in presymptomatic Alzheimer’s disease. Nat Med.

[26] Weston PSJ, Poole T, Ryan NS, Nair A, Liang Y, Macpherson K (2017). Serum neurofilament light in familial Alzheimer disease: a marker of early neurodegeneration. Neurology..

[27] Oeckl P, Halbgebauer S, Anderl-Straub S, Steinacker P, Huss AM, Neugebauer H (2019). Glial fibrillary acidic protein in serum is increased in Alzheimer’s disease and correlates with cognitive impairment. J Alzheimers Dis.

[28] Ishiki A, Kamada M, Kawamura Y, Terao C, Shimoda F, Tomita N (2016). Glial fibrillar acidic protein in the cerebrospinal fluid of Alzheimer’s disease, dementia with Lewy bodies, and frontotemporal lobar degeneration. J Neurochem.

[29] Fukuyama R, Izumoto T, Fushiki S (2001). The cerebrospinal fluid level of glial fibrillary acidic protein is increased in cerebrospinal fluid from Alzheimer’s disease patients and correlates with severity of dementia. Eur Neurol.

[30] Jesse S, Steinacker P, Cepek L, von Arnim CA, Tumani H, Lehnert S (2009). Glial fibrillary acidic protein and protein S-100B: different concentration pattern of glial proteins in cerebrospinal fluid of patients with Alzheimer’s disease and Creutzfeldt-Jakob disease. J Alzheimers Dis.

[31] Abu-Rumeileh S, Steinacker P, Polischi B, Mammana A, Bartoletti-Stella A, Oeckl P (2019). CSF biomarkers of neuroinflammation in distinct forms and subtypes of neurodegenerative dementia. Alzheimers Res Ther.

[32] Nagele RG, D’Andrea MR, Lee H, Venkataraman V, Wang HY (2003). Astrocytes accumulate A beta 42 and give rise to astrocytic amyloid plaques in Alzheimer disease brains. Brain Res.

[33] Simpson JE, Ince PG, Lace G, Forster G, Shaw PJ, Matthews F (2010). Astrocyte phenotype in relation to Alzheimer-type pathology in the ageing brain. Neurobiol Aging.

[34] Garwood CJ, Ratcliffe LE, Simpson JE, Heath PR, Ince PG, Wharton SB (2017). Review: astrocytes in Alzheimer’s disease and other age-associated dementias: a supporting player with a central role. Neuropathol Appl Neurobiol.

[35] Hondius DC, van Nierop P, Li KW, Hoozemans JJ, van der Schors RC, van Haastert ES (2016). Profiling the human hippocampal proteome at all pathologic stages of Alzheimer’s disease. Alzheimers Dement.

[36] van der Flier WM, Pijnenburg YA, Prins N, Lemstra AW, Bouwman FH, Teunissen CE (2014). Optimizing patient care and research: the Amsterdam dementia cohort. J Alzheimers Dis.

[37] van der Flier WM, Scheltens P (2018). Amsterdam dementia cohort: performing research to optimize care. J Alzheimers Dis.

[38] Jessen F, Amariglio RE, van Boxtel M, Breteler M, Ceccaldi M, Chetelat G (2014). A conceptual framework for research on subjective cognitive decline in preclinical Alzheimer’s disease. Alzheimers Dement.

[39] Petersen RC, Smith GE, Waring SC, Ivnik RJ, Tangalos EG, Kokmen E (1999). Mild cognitive impairment: clinical characterization and outcome. Arch Neurol.

[40] Albert MS, DeKosky ST, Dickson D, Dubois B, Feldman HH, Fox NC (2011). The diagnosis of mild cognitive impairment due to Alzheimer’s disease: recommendations from the National Institute on Aging-Alzheimer’s Association workgroups on diagnostic guidelines for Alzheimer’s disease. Alzheimers Dement.

[41] McKhann G, Drachman D, Folstein M, Katzman R, Price D, Stadlan EM (1984). Clinical diagnosis of Alzheimer’s disease: report of the NINCDS-ADRDA Work Group under the auspices of Department of Health and Human Services Task Force on Alzheimer’s Disease. Neurology..

[42] McKhann GM, Knopman DS, Chertkow H, Hyman BT, Jack CR, Kawas CH (2011). The diagnosis of dementia due to Alzheimer’s disease: recommendations from the National Institute on Aging-Alzheimer’s Association workgroups on diagnostic guidelines for Alzheimer’s disease. Alzheimers Dement.

[43] Scheltens P, Leys D, Barkhof F, Huglo D, Weinstein HC, Vermersch P (1992). Atrophy of medial temporal lobes on MRI in “probable” Alzheimer’s disease and normal ageing: diagnostic value and neuropsychological correlates. J Neurol Neurosurg Psychiatry.

[44] de Wilde A, Reimand J, Teunissen CE, Zwan M, Windhorst AD, Boellaard R (2019). Discordant amyloid-beta PET and CSF biomarkers and its clinical consequences. Alzheimers Res Ther.

[45] Zwan MD, Bouwman FH, Konijnenberg E, van der Flier WM, Lammertsma AA, Verhey FR (2017). Diagnostic impact of [(18)F]flutemetamol PET in early-onset dementia. Alzheimers Res Ther.

[46] Slot RER, Verfaillie SCJ, Overbeek JM, Timmers T, Wesselman LMP, Teunissen CE (2018). Subjective Cognitive Impairment Cohort (SCIENCe): study design and first results. Alzheimers Res Ther.

[47] Ossenkoppele R, van der Flier WM, Verfaillie SC, Vrenken H, Versteeg A, van Schijndel RA (2014). Long-term effects of amyloid, hypometabolism, and atrophy on neuropsychological functions. Neurology..

[48] de Wilde A, van der Flier WM, Pelkmans W, Bouwman F, Verwer J, Groot C (2018). Association of amyloid positron emission tomography with changes in diagnosis and patient treatment in an unselected memory clinic cohort: the ABIDE project. JAMA Neurol..

[49] Johnson-Wood K, Lee M, Motter R, Hu K, Gordon G, Barbour R (1997). Amyloid precursor protein processing and A beta42 deposition in a transgenic mouse model of Alzheimer disease. Proc Natl Acad Sci U S A.

[50] Verhage F (1965). Intelligence and age in a Dutch sample. Hum Dev.

[51] Liddelow SA, Guttenplan KA, Clarke LE, Bennett FC, Bohlen CJ, Schirmer L (2017). Neurotoxic reactive astrocytes are induced by activated microglia. Nature..

[52] Lewczuk P, Gaignaux A, Kofanova O, Ermann N, Betsou F, Brandner S (2018). Interlaboratory proficiency processing scheme in CSF aliquoting: implementation and assessment based on biomarkers of Alzheimer’s disease. Alzheimers Res Ther.

[53] Rozga M, Bittner T, Batrla R, Karl J (2019). Preanalytical sample handling recommendations for Alzheimer’s disease plasma biomarkers. Alzheimers Dement (Amst).

[54] Vanderstichele H, Bibl M, Engelborghs S, Le Bastard N, Lewczuk P, Molinuevo JL (2012). Standardization of preanalytical aspects of cerebrospinal fluid biomarker testing for Alzheimer’s disease diagnosis: a consensus paper from the Alzheimer’s Biomarkers Standardization Initiative. Alzheimers Dement.

[55] Mielke MM, Syrjanen JA, Blennow K, Zetterberg H, Vemuri P, Skoog I (2019). Plasma and CSF neurofilament light: relation to longitudinal neuroimaging and cognitive measures. Neurology..

[56] Mielke MM, Hagen CE, Xu J, Chai X, Vemuri P, Lowe VJ (2018). Plasma phospho-tau181 increases with Alzheimer’s disease clinical severity and is associated with tau- and amyloid-positron emission tomography. Alzheimers Dement.

[57] Thijssen EH, La Joie R, Wolf A, Strom A, Wang P, Iaccarino L, et al. Diagnostic value of plasma phosphorylated tau181 in Alzheimer’s disease and frontotemporal lobar degeneration. Nat Med. 2020;26(3):387–97. 10.1038/s41591-020-0762-2 PMC7101073 32123386

[58] Ebenau JL, Timmers T, Wesselman LMP, Verberk IMW, Verfaillie SCJ, Slot RER, et al. ATN classification and clinical progression in subjective cognitive decline: the SCIENCe project. Neurology. 2020;95(1):e46–58. 10.1212/WNL.0000000000009724 PMC7371376 32522798

