# Supplementary material for: Combination of plasma amyloid beta(1-42/1-40) and glial fibrillary acidic protein strongly associates with cerebral amyloid pathology
Source: Alzheimers Res Ther. 2020 Sep 28;12:118. doi: 10.1186/s13195-020-00682-7 (PMC7523295; doi:10.1186/s13195-020-00682-7)
Supplement: Supplementary file 1 — Additional file 1: Supplementary Table 1. Demographics, clinical characteristics, and plasma marker concentrations of the study population stratified by syndrome diagnosis. Supplementary figure 1. Heat plots with predicted probabilities for amyloid PET positivity in the non-demented subset. [file 13195_2020_682_MOESM1_ESM.docx]

**SUPPLEMENTARY FILE**

**Supplementary table 1** Demographics, clinical characteristics, and plasma marker concentrations of the study population stratified by syndrome diagnosis.

|  | **SCD** | **MCI** | **AD-dementia** |
| --- | --- | --- | --- |
|  | ***n=70*** | ***n=50*** | ***n=132*** |
| Age | 62 ± 9 | 66 ± 8 | 63 ± 7 |
| Female sex | 33 (47%) | 14 (28%) | 67 (51%) |
| Education | 5.4 ± 1.3 | 5.6 ± 1.2 | 5.2 ± 1.1 |
| Amyloid PET positivity | 18 (26%) | 26 (52%) | 132 (100%) |
| APOE ε4 carriership | 23 (33%) | 27 (54%) | 84 (64%) |
| MMSE | 28 ± 2 | 26 ± 2 | 22 ± 4 |
| MTA score | 0 (0 – 0) | 1 (0 – 1.5) | 1 (0.5 – 1.5) |
|  |  |  |  |
| Plasma Abeta_(1-42/1-40)_ | 0.16 ± 0.04 | 0.15 ± 0.04 | 0.14 ± 0.03 |
| Plasma GFAP, pg/mL | 105 ± 65 | 130 ± 59 | 176 ± 79 |
| Plasma NfL, pg/mL | 10 ± 4 | 14 ± 6 | 16 ± 11 |

*Baseline demographic features of the study population stratified for syndrome diagnosis. Data is presented as mean* ± SD, median (25^th^ – 75^th^ percentile) or n (%). *Education scoring is according to the Verhage (1965) system with a scale ranging from 1 to 7*. *APOE status was available for n=244, MTA score (average of right and left) was available for n=182, plasma Abeta_(1-42/1-40)_, plasma GFAP for n=247 and plasma NfL for n=251. SCD = subjective cognitive decline, MCI = mild cognitive impairment, AD = Alzheimer’s disease, PET=Positron Emission Tomography, APOE = apolipoprotein E, MMSE = mini mental state examination, MTA = medial temporal lobe atrophy, Abeta = amyloid beta, GFAP = Glial fibrillary acidic protein, NfL = Neurofilament light.*

**Supplementary figure 1. Heat plots with predicted probabilities for amyloid PET positivity in the non-demented subset**

**
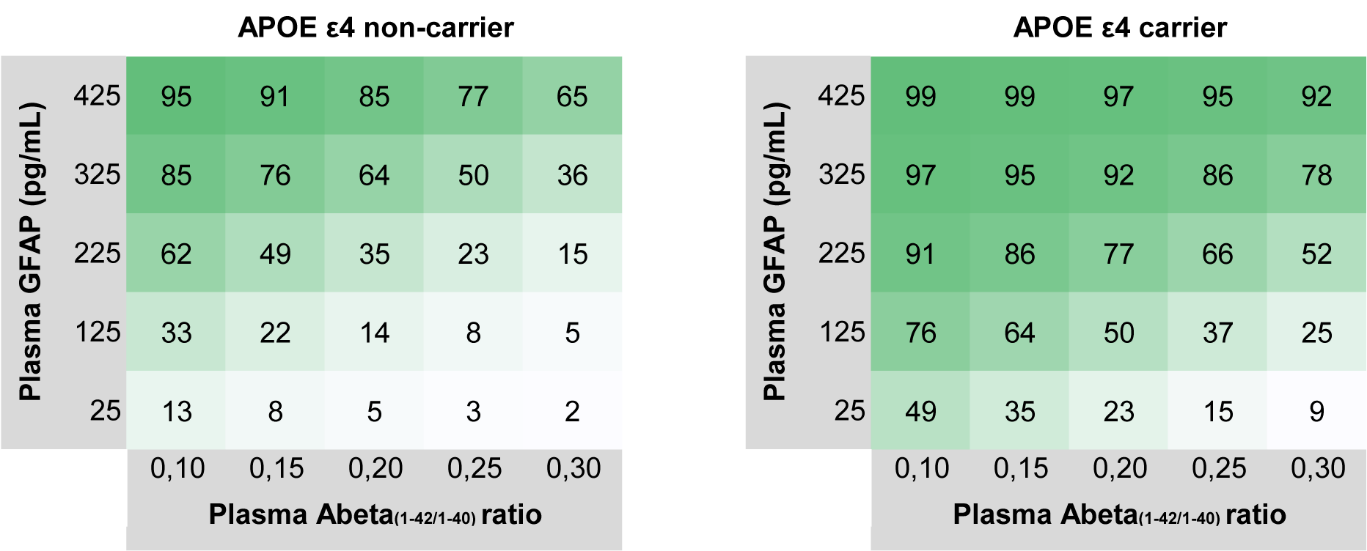
**

*Heat plots were constructed by filling out the logistic regression formula of the non-demented subset (SCD + MCI) with constant=-1.053, and beta’s B=-11.369 for Abeta_(1-42/1-40)_, B=0.012 for GFAP and B=1.845 for APOE ε4 carriership (non-carrier=0, carrier=1). Abeta=amyloid beta, GFAP = Glial fibrillary acidic protein, APOE = Apolipoprotein E.*
